# Transient refractory period in severe iodinated contrast media allergy: a case report

**DOI:** 10.1186/s13223-025-00972-5

**Published:** 2025-10-02

**Authors:** Lara S. Dungan, Fionnuala Cox

**Affiliations:** 1 Beaumont Hospital, Beaumont Road, Dublin 9, Ireland; 2 Royal College of Surgeons in Ireland, Dublin, Ireland

**Keywords:** Iodinated contrast media, Hypersensitivity reaction, Anaphylaxis, Refractory period, Skin testing, Contrast allergy

## Abstract

**Background:**

Hypersensitivity reactions to iodinated contrast media (ICM) are rare but can be life-threatening. Management typically involves avoidance of the offending agent and the use of alternative imaging strategies. The phenomenon of a transient refractory period—wherein a patient does not exhibit an allergic response upon re-exposure to the allergen shortly after an initial reaction—has been proposed but is not well-documented in the context of ICM.

**Case presentation:**

We report the case of a 56-year-old woman who experienced an anaphylaxis associated cardiac arrest following administration of iopamidol 370 (Niopam 370) during a computed tomography pulmonary angiogram (CTPA). She was resuscitated, intubated, and stabilized with noradrenaline. Two hours later, she underwent a second CT scan using iopamidol 300 (Niopam 300) without any obvious immediate hypersensitivity reaction. Subsequent skin testing was positive for both Niopam 370 and Niopam 300, but negative for alternative agents - iodixanol (Visipaque) and iohexol (Omnipaque).

**Conclusions:**

This case suggests the presence of a transient refractory period following a severe hypersensitivity reaction to ICM, during which re-exposure to the allergen does not elicit an immediate response. Understanding this phenomenon could have significant implications for the management of urgent imaging needs in patients with known ICM hypersensitivity.

## Background

Iodinated contrast media (ICM) are essential for enhanced diagnostic imaging, particularly in computed tomography (CT) studies. While ICM are generally well-tolerated, immediate hypersensitivity reactions can occur in approximately 0.7% of non-ionic, low-osmolality contrast exposures [1]. Severe reactions such as anaphylaxis are much rarer, but potentially fatal [1].

The management of such reactions involves immediate resuscitation, avoidance of the suspected agent in the future, and consideration of premedication or substitution with alternative contrast agents [2]. Skin testing may assist in identifying the causative agent and safe alternatives [3].

The concept of a transient “refractory period” following anaphylaxis, during which a patient may not respond to subsequent exposure to the same allergen, has been suggested in allergic and immunologic literature [4]. However, it is poorly characterized in relation to ICM exposure. This case explores such a phenomenon, presenting clinical evidence to support its possible existence in the setting of ICM hypersensitivity.

## Case presentation

We describe the case of a 56 year old female who was admitted to hospital with a one month history of shortness of breath, with bilateral lower limb and abdominal swelling. She had a history of chronic obstructive pulmonary disease (COPD) and asthma, migraines, hypertension, hyperlipidaemia, peripheral vascular disease, with a middle cerebral artery clipping and prior pulmonary embolism in the setting of COVID-19 infection. Patient underwent a CT pulmonary angiogram (CTPA) for investigation of her acute presentation and received 40 ml of iopamidol 370 (Niopam 370) as an ICM. She had no previous history of drug allergy. She had received iopamidol 370 on at least four occasions in the previous six years and had most recently tolerated it 31 months prior to this exposure.

Within five minutes of completing the CTPA she became acutely unwell and had a cardiac arrest with a ventricular tachycardia noted. Angioedema and urticaria were not noted. She had a successful resuscitation attempt and was subsequently intubated and her blood pressure was maintained on an adrenaline infusion in the ICU with a mean arterial pressure over seventy as a target. She was administered 100 mg of IV hydrocortisone in the immediate post-arrest period but was given no antihistamine. Despite an initial concern that the cardiac arrest could have been caused by anaphylaxis, the medical team decided to perform a subsequent CT two hours and eighteen minutes after the initial CT, using 80mls of iopamidol 300 (Niopam 300) as the patient remained hyperkalaemic with a profound metabolic lactic acidosis. Iopamidol 300 contains the same active ingredient as iopamidol 370. She remained stable throughout the second CT scan, albeit while on an adrenaline infusion.

**Fig. 1:**
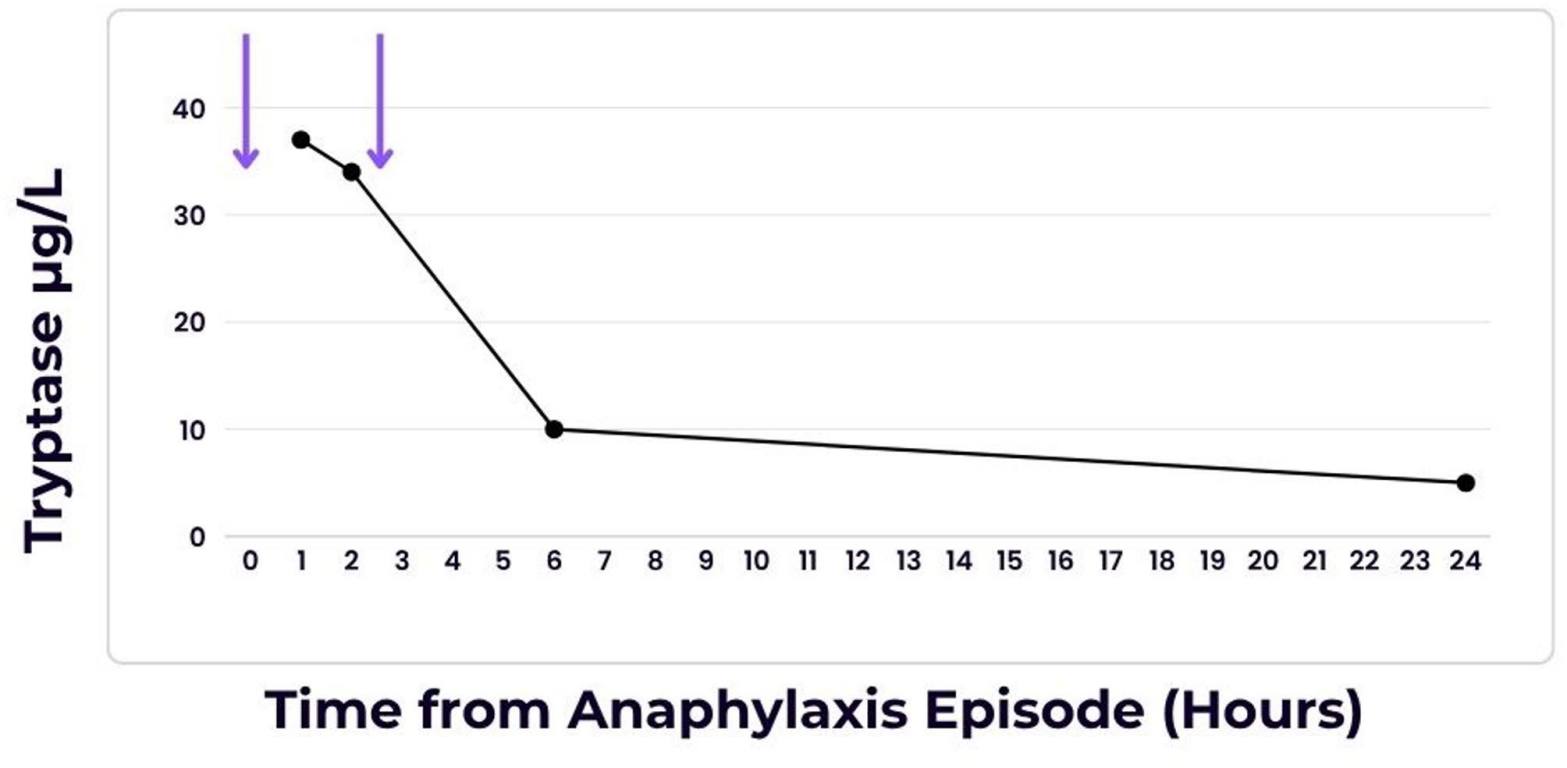
Serum Tryptase versus Time from Anaphylaxis Episode. Purple arrows represent the two time points at which iodinated contrast media was administered

Mast cell tryptase was sent and was found to be raised to 37 µg/L (upper limit of normal 14 µg/L) at the time of the event, 34 µg/L at two hours, 10 µg/L at six hours and demonstrated a normal baseline of 5 µg/L at 24 h (Fig. 1). Skin testing was performed six weeks after the event. Skin prick testing was performed to undiluted contrast media and intradermal testing was performed at 1:10 dilutions in line with the Australia and New Zealand Anaesthetic Allergy Group (ANZAAG) [5]. Intradermal testing was positive with iopamidol 300 and iopamidol 370 but negative with iohexol (omnipaque) and iodixanol (visipaque), two alternative ICM. The patient did not consent to challenge with iohexol or iodixanol. She has not had any subsequent exposure with any iodinated contrast media.

## Discussion and conclusions

This case presents an unusual scenario where a patient demonstrated a severe, life-threatening reaction to iopamidol, followed by a second uneventful exposure to a closely related formulation of the same compound within a short time window, albeit while her blood pressure was being maintained on an adrenaline infusion. Positive skin tests confirmed sensitization to both formulations, suggesting that the lack of response during the second exposure was not due to absence of hypersensitivity, but may have been due to a temporary modulation of the immune response. Additionally, it should be noted that this patient was on an adrenaline infusion and adrenaline has been demonstrated to stabilise mast cells so it may have contributed to blocking mast cell degranulation [6].

The concept of a transient “refractory period” following anaphylaxis has been observed in immunologic studies, where a short-term suppression or depletion of mast cell and basophil activity may occur after a major degranulation event [4, 7]. It is plausible that this physiological state offers a temporary window during which further allergic responses are attenuated or absent, even if the sensitizing antigen is reintroduced. The fact that the patient tolerated ICM in this theoretical refractory period could perhaps have given false reassurance that the initial cause of cardiac arrest was not anaphylaxis, however in this case due diligence was done and the patient was referred to immunology services for evaluation. In addition, the dynamic rise in tryptase gave good supportive evidence that assisted with the diagnosis. If anaphylaxis is suspected, patients should be referred to the local immunology and allergy service for further work up and evaluation. There are, of course, non-IgE mediated mechanisms by which ICM can induce allergic-type symptoms including direct mast cell and basophil degranulation, however this is more common with older high-osmolarity ionic agents [8].

Although described in the literature as a theoretical concern, there is limited clinical documentation of this phenomenon in the context of iodinated contrast agents. Different groups have outlined guidelines for skin testing and re-exposure, but do not explicitly address the possibility of a refractory window [3, 9]. Thomsen et al. have described desensitization protocols and contrast allergy management but again without specific reference to acute immune refractoriness [10].

While this case offers rare clinical insight into such a phenomenon, it must be emphasized that re-administration of contrast media after a hypersensitivity reaction is not recommended outside of life-threatening or unavoidable clinical situations. More research is needed to validate the presence, duration, and mechanism of this refractory period in ICM hypersensitivity.

## Data Availability

No datasets were generated or analysed during the current study.

## Abbreviations

ICM: Iodinated contrast media
CTPA: Computed tomography pulmonary angiogram
SPT: Skin prick test
IDT: Intradermal test
COPD: Chronic obstructive pulmonary disease
